# This newly recognized coronavirus makes one wonder why we were so unprepared

**DOI:** 10.3325/cmj.2020.61.201

**Published:** 2020-04

**Authors:** Charles H. Calisher

When I worked at the US Centers for Disease Control and Prevention we called them “the diseases of the week,” diseases, newly recognized or reappearing, that suddenly appeared in the United States or elsewhere in the world. That happened more often than one would expect, but should not have surprised us. Diseases of all sorts have been recognized for thousands of years and, recently it seems, more often. Why? Do the media make more of an outbreak than the outbreak deserves? Is the particular situation (location, host, disease, peculiarity of transmission) of sufficient fascination that everyone wants to know “What is this thing?” or is someone, or more than someone, benefitting (money, politics, reputation) from the media frenzy or want to become famous? When someone dies in a place far from your home and that death does not appear to threaten you or your family, do we think to ourselves that it is not our problem? Given the current exceedingly large human population, the commonality of air travel, and the frequent eruption of wars, civil and otherwise, and the attendant movement of refugees, it is a problem, or a potential problem for people everywhere, as the discovery of this new coronavirus disease has demonstrated for all to recognize.

The coronaviruses (named for the “crown” [corona] formed by their surface protein spikes when viewed by electron microscopy) comprise a large family of viruses, most of which usually cause mild to moderate upper-respiratory tract illnesses, such as the common cold, in people. There are hundreds of known coronaviruses; most circulate among pigs, camels, bats, and cats. Sometimes those viruses jump to humans (a “spillover” event) and cause disease in them. Seven coronaviruses currently are known to cause human disease. Infections with four of these are mild but three coronaviruses can cause more serious illnesses in humans: severe acute respiratory syndrome (SARS) coronavirus (SARS-CoV), which was first recognized in 2002 in China and disappeared two years later; Middle East respiratory syndrome (MERS) coronavirus, which was first recognized in 2012 and remains in circulation in camels, mostly in Saudi Arabia; and this recent nightmare virus, causing what has been named by the World Health Organization COVID-19 (disease name), which emerged in late 2019 in China (*https://www.who.int/csr/don/05-january-2020-pneumonia-of-unkown-cause-china/en/)* and now is pandemic. Using increasingly informed models (*https://www.sciencemag.org/news/2020/03/mathematics-life-and-death-how-disease-models-shape-national-shutdowns-and-other*), a global effort now is under way to contain its spread. COVID-19 is caused by the coronavirus known as SARS-CoV-2 (*https://talk.ictvonline.org/*). Its containment has not been completely successful anywhere and only partially and belatedly successful even in China, which used heroic and authoritarian means in its efforts to control the virus. The virus appears to have been well controlled but not eradicated from Hubei Province where it was discovered, and perhaps where it originated in bats (1,2).

I must admit that my experience with coronaviruses is limited. My doctoral research was with mouse hepatitis virus, now classified by the International Committee on the Taxonomy of Viruses as a virus in the species *Murine coronavirus*, genus *Betacoronavirus*, subfamily *Coronavirinae*, family *Coronaviridae* (3). Anyway, it is a coronavirus, one of many, and the SARS-CoV-2 virus is “a” coronavirus not “the” coronavirus” or “coronavirus.”

My experience with another coronavirus was not a happy one. Many years ago, I headed to Bethesda, Maryland, to attend a meeting at the US National Institutes of Health (NIH) and to deliver a packet of salary checks, which had been sent in error to our laboratory in Colorado. When I arrived in Bethesda late in the day, I felt ill and decided to just go to bed in my motel, rather than to dinner. The next morning, I was awakened by someone banging on the door of my room. I opened the door to see two US Government enforcement officers demanding that I turn over the salary packet to them. After they showed me their identifications, I gave them the checks and went back to bed, realizing that I had a severe headache, a fever, and myalgias. I called my sponsor at the NIH, told him what was happening, and asked to be excused from the meeting. He told me that I should report to the Clinical Center, a research hospital on the NIH campus. They took samples from me and insisted that I stay overnight, after which I returned home, still slightly ill. A few days later, a telephone caller from NIH informed me that they had isolated and identified a coronavirus from my throat swab sample. They did not identify the specific virus because of the characterization of my illness and because coronaviruses were and are so common.

Other experiences I had were more enjoyable and educational. When we realized that bats had been studied regarding rabies virus, but not much else regarding other viruses, I helped author a paper that reviewed the few known occurrences of viruses of bats (4), an extremely popular publication, I am pleased to say. Simply for my own interest, I accompanied experienced chiropterologists on bat-trapping and sampling expeditions at sites they had been studying previously and subsequently accompanied researchers of the US Geological Survey who intended to teach me even more about capturing bats. We waited until dark, set up the capture apparatuses, and processed the bats that had been captured; fascinating. Remarkably, from a very few bats, we detected two different coronaviruses. I thought that would be my last association with bats and coronaviruses, but was once again wrong.

## Discovery of a pandemic coronavirus

In late 2019 the Chinese government notified the World Health Organization that it had detected a hitherto unrecognized coronavirus (now named SARS-CoV-2) in people with respiratory distress; some had died and the disease was reported to be spreading (*https://www.who.int/csr/don/05-january-2020-pneumonia-of-unkown-cause-china/en*/). Of course, the disease was not spreading, it was the virus that causes the disease that was spreading – and frighteningly fast, and far. It was obvious then that this might become a global problem, and it has become just that, a pandemic. The government of China and, in particular, the Chinese investigators studying this problem, deserve recognition and praise for the courageous work they did in the early days of this extraordinary event.

Although SARS-CoV-2 is related to SARS-CoV, the causative agent of SARS, it differs sufficiently in many ways, enough to give it a different name and to consider it a very different virus. Its rapid spread, essentially world-wide, now threatens to cause thousands, if not millions, of fatalities globally. At the time of this writing we cannot see over the horizon to its end. Perhaps some plant extract, existing or newly formulated anti-viral drug, or other developed substance will be found to be effective in treating the disease caused by SARS-CoV-2. More likely, it will take an effective and safe vaccine to end this horror. That will not be simple and straightforward.

## Illness and epidemiology

Early observations of patients suffering from infection with this virus suggested that young people were not sickened by it, old people were killed by it, and those in between became sick but recovered relatively quickly from infection with it. It was not long before this supposed relatively simple clinical picture was seen to be incorrect. Now the epidemiologic observations regarding a larger number of patients indicate that the death rate in males is greater than the death rate in females, that older people with co-morbidities are more likely to die than are otherwise healthy people. Also, young people can be infected with SARS-CoV-2, albeit with a lower case-fatality rate, but also shed virus, and older patients can become ill and shed virus (for how long and by what routes?). Critically, people in all age groups may become infected with the virus but some of them may shed virus yet do not become ill. It is important to recognize that all people not infected with SARS-CoV-2 themselves comprise a susceptible population, a harbinger of the next round of illnesses caused by this virus. This is a frightening potential situation and one suggesting that total elimination of SARS-CoV-2 is unlikely. Thus, what will occur when the personal controls we humans have self-applied are considered no longer necessary? Will the populace simply be relieved and wander out of their houses to resume life as before? It is likely that life as we knew it will not be as it was before this disease was recognized. There will be no miracle that occurs to make all surfaces safe on “Day One.”

In summary, SARS-CoV-2 has led to a disastrous situation and one with which the medical community of each nation must manage in some way, perhaps through World Health Organization protocols, perhaps by a novel approach. Because humans infected with SARS-CoV-2 may shed virus before they become ill, or even if they never become ill, they may serve as reservoirs of the virus. This is a principal reason for patients and non-patients alike not to contact anyone other than a house-mate (spouse, children, and anyone else sharing living quarters) for two weeks or longer. This is called “social distancing” but it is actually “physical distancing” because computers, telephones, the mail system, and many other tools allow us to socialize at a distance. Rumors in the popular press have not helped the public understand the situation, and statements by politicians, as always, bend the truth either intentionally or because they do not understand what the truth is.

## Molecular effects complicate our understanding of the epidemiology of this virus

The spike proteins of coronaviruses mediate viral entry into cells by binding to a receptor on the host cell surface and then fusing viral and host membranes (5). However, preliminary evidence suggests that in some instances IgG-virus complexes may make possible the entry of viruses to cells and infection of those cells by what has been called antibody-dependent enhancement, in so doing increasing severity of infection (6,7).

This phenomenon has been shown to occur in infections with feline coronavirus, SARS-CoV, MERS coronavirus, influenzaviruses, Zika virus, human immunodeficiency virus, and dengue viruses, and probably occurs with other viral infections, or after certain vaccinations. More relevant to the current pandemic, it may be that older patients produce high affinity IgG antibody and younger patients do not, thus older patients might very well not respond as desired to a vaccine, treatment with monoclonal antibody, or immune serum transfer. As older patients are the ones at higher risk of deadly outcome, vaccination might not be suitable in this instance. I hope I am wrong about this.

## George Santayana: “Those who cannot remember the past are condemned to repeat it.”

So, was the SARS experience just “the disease of the week” or month or year? It was discovered in 2003 and disappeared after 2004, after it had spread from Asia to North America, South America, and Europe. As is typical, funding to support research on this disease declined precipitously. That is also typically unfortunate, as it is only research that can maintain and defend preparedness. Huge amounts of money have been spent to gather information we hoped would lead to the prediction of outbreaks. Obviously, we have accomplished little in this regard, a lesson in itself. But have we learned nothing? Perhaps each country, wealthy or poor, should consider putting some proportion of its income into a fund put aside as a contingency in the event of an (inevitable) emergency. That such a fund might have to come from additional taxes is no reason to ignore this possibility; pay it now or pay it later – or pay the consequences. Governments that do not protect their citizens from diseases are simply criminal enterprises.

As humans continue to plunder the natural world by using such extractive sources, such as coal, oil, and other forms of non-renewable energy, we scavenge in unexplored areas containing wildlife that likely are infected with presently unrecognized viruses that are potential pathogens for humans and/or livestock and/or other wildlife. Recent cross-species transmission events, particularly those jumping from wildlife infected with genetically malleable RNA viruses have been shown to be the primary source of recently recognized human pathogens (8).

How many more potential human pathogens are there still, and thankfully, unrecognized? What do we do with the possibly predictive information we gather when a previously unknown virus is recognized? Do we continue to repeat over and over again our discoveries and our errors? Are we, as a species, committing suicide?

Note: At the time this opinionated column was written, at least 1 000 000 people have been infected with SARS-CoV-2 and nearly 50 000 have died. China, Italy, Spain, and the USA have suffered most. The end is not in sight and the devastation this virus has caused will undoubtedly lead not only to human physical misery and great sadness but to economic turmoil.

## References

[1] Menachery VD, Yount BL, Debbink K, Agnihothram S, Gralinski LE, Plante JA (2015). A SARS-like cluster of circulating bat coronaviruses shows potential for human emergence.. Nat Med.

[2] Andersen KG, Rambaut A, Lipkin WI, Holmes EC, Garry RF (2020). The proximal origin of SARS-CoV-2.. Nat Med.

[3] King AMQ, Adams MJ, Carstens EB, Lefkowitz EJ, editors. Virus taxonomy: classification and nomenclature of viruses. Ninth report of the international committee on taxonomy of viruses. International Union of Microbiological Societies, Virology Division, Elsevier Academic Press, San Diego California, USA; 2012.

[4] Calisher CH, Childs JE, Field HE, Holmes KV, Schountz T (2006). Bats: important reservoir hosts of emerging viruses.. Clin Microbiol Rev.

[5] Wan Y, Shang J, Sun S, Tai W, Chen J, Geng Q (2020). Molecular mechanism for antibody-dependent enhancement of coronavirus entry.. J Virol.

[6] Wang SF, Tseng SP, Yen CH, Yang JY, Tsao CH, Shen CW (2014). Antibody-dependent SARS coronavirus infection is mediated by antibodies against spike proteins.. Biochem Biophys Res Commun.

[7] Tetro JA (2020). Is COVID-19 receiving ADE from other coronaviruses?. Microbes Infect.

[8] Parrish CR, Holmes EC, Morens DM, Park E-C, Burke DS, Calisher CH (2008). Cross-species viral transmission and the emergence of new epidemic diseases.. Microbiol Mol Biol Rev.

